# Latent Phase Identification of High-Frequency Micro-Scale Gamma Spike Transients in the Hypoxic Ischemic EEG of Preterm Fetal Sheep Using Spectral Analysis and Fuzzy Classifiers

**DOI:** 10.3390/s20051424

**Published:** 2020-03-05

**Authors:** Hamid Abbasi, Alistair J. Gunn, Laura Bennet, Charles P. Unsworth

**Affiliations:** 1 Department of Engineering Science, Faculty of Engineering, University of Auckland, Auckland 1142, New Zealand; c.unsworth@auckland.ac.nz; 2 Department of Physiology, Faculty of Medical and Health Sciences, University of Auckland, Auckland 1023, New Zealand; aj.gunn@auckland.ac.nz (A.J.G.); l.bennet@auckland.ac.nz (L.B.)

**Keywords:** hypoxic–ischemic encephalopathy (HIE), automatic detection and quantification, high-frequency micro-scale gamma spikes, spectral Fourier transform analysis, fuzzy, electroencephalogram (EEG), electrocorticogram (ECoG)

## Abstract

Premature babies are at high risk of serious neurodevelopmental disabilities, which in many cases are related to perinatal hypoxic–ischemic encephalopathy (HIE). Studies of neuroprotection in animal models consistently suggest that treatment must be started as early as possible in the first 6 h after hypoxia–ischemia (HI), the so-called latent phase before secondary deterioration, to improve outcomes. We have shown in preterm sheep that EEG biomarkers of injury, in the form of high-frequency micro-scale spike transients, develop and evolve in this critical latent phase after severe asphyxia. Real-time automatic identification of such events is important for the early and accurate detection of HI injury, so that the right treatment can be implemented at the right time. We have previously reported successful strategies for accurate identification of EEG patterns after HI. In this study, we report an alternative high-performance approach based on the fusion of spectral Fourier analysis and Type-I fuzzy classifiers (FFT-Type-I-FLC). We assessed its performance in over 2520 min of latent phase EEG recordings from seven asphyxiated in utero preterm fetal sheep exposed to a range of different occlusion periods. The FFT-Type-I-FLC classifier demonstrated 98.9 ± 1.0% accuracy for identification of high-frequency spike transients in the gamma frequency band (namely 80–120 Hz) post-HI. The spectral-based approach (FFT-Type-I-FLC classifier) has similar accuracy to our previous reverse biorthogonal wavelets rbio2.8 basis function and type-1 fuzzy classifier (rbio-WT-Type-1-FLC), providing competitive performance (within the margin of error: 0.89%), but it is computationally simpler and would be readily adapted to identify other potentially relevant EEG waveforms.

## 1. Introduction

Newborns with signs of hypoxic–ischemic encephalopathy (HIE) are critically at risk of developing lifelong debilitating conditions such as cognitive disorders, epilepsy, and cerebral palsy [1,2,3]. An HI event, is associated with profound suppression of EEG activity. After the period of HI, EEG activity progressively recovers along with restoration of cerebral metabolism, during a latent phase of recovery for approximately 6 h [4,5,6,7,8]. The progressive failure of oxidative metabolism from the end of the latent phase causes subcortical neural cells to die over hours to days during a secondary phase lasting 48–72 h [4,5,6,7,8,9]. The secondary phase is characterized by the appearance of high-amplitude stereotypic evolving seizures in the EEG recordings. Finally, the brain starts to rebuild its architecture during a tertiary phase [4,5,6,7,8,9,10]. Using fetal sheep models, the pioneers in our team, Gunn and Bennet, have demonstrated that the latent phase is the key time period that provides the optimal opportunity to initiate neuroprotective treatment before it is too late [2,7,11,12].

At present, we lack biological markers (biomarkers) that reveal timing information or allow determining the severity of HIE so that clinicians could determine whether there is still the opportunity to initiate neuroprotective protocols or not [1,2,11]. Moreover, currently, there is no available established EEG technique to indicate if an hypoxia–ischemia (HI) insult has occurred or not [5,13]. Using fetal sheep models, we have demonstrated that EEG biomarkers of HIE emerge temporarily in the form of micro-scale transients in the signal during the latent phase, after an acute-HI insult. [5,6,7,8,14,15,16,17,18]. The micro-scale EEG transients are mainly categorized as spikes, sharps, slow waves, and complexes, as well as ongoing stereotypic evolving micro-scale seizures (SEMS) [5,6,7,8,9,10,11,14,15,16,19] (see Figure 2 of ref [20]). Early automatic identification of these transients could support in-time diagnosis of at-risk infants and increase the chances of effective treatment before the window of opportunity is closed. 

**Figure 1:**
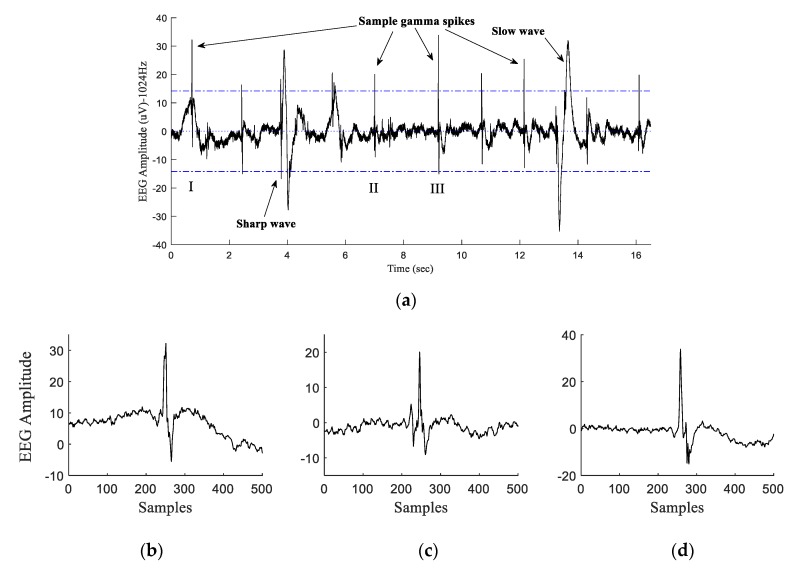
(**a**) A sample 16-sec electroencephalogram (EEG)/electrocorticogram (ECoG) recorded at 1024 Hz, 90 min post-hypoxic–ischemic (HI) insult containing gamma spikes, a sharp wave, and a slow wave. (**b**—**d**) represent the (I), (II), and (III) sample labelled HI spike transients.

We have recently developed automated algorithms for the accurate identification of HI spikes and sharp waves [16,17,18,20,21] and demonstrated that an increase in the number of automatically identified micro-scale sharp waves from 2–6 h after HI is associated with worse fetal outcomes [9,10,21]. Monitoring of post-asphyxial EEG from instrumented preterm fetal sheep demonstrates that micro-scale HI spike transients emerge in the gamma frequency band (namely 80–120 Hz) along the 1024 Hz high-frequency sampled electrocorticogram (ECoG) within the latent phase (Figure 1). Clinical and experimental epilepsy studies have demonstrated that bursts of high-frequency EEG transients/oscillations (HFOs), with the frequency of >80 Hz, during short time intervals help to predict the onset of later epileptic seizures that occur with higher amplitudes [22,23]. Our team has previously investigated the capabilities of different techniques for automatic identification of HI spike transients within the low-resolution EEG (sampling frequency: 64 Hz) using Short-Time Fourier Transform (STFT) [24], Haar continuous wavelet transform (CWT) [25], and Type-2 fuzzy classifiers [26]. Data acquisition at higher sampling frequencies has allowed us to capture detailed EEG/ECoG at higher 1024 Hz resolutions.

Using 1024 Hz sampled ECoGs from seven in utero preterm fetal sheep, we recently demonstrated that the fusion of thresholded CWT (using the reverse biorthogonal wavelets rbio2.8 basis function) and type-1 fuzzy classifier (rbio-WT-Type-1-FLC) significantly improves the accuracy of the spike identification in real time [20]. Using data from a similar database, this paper represents a new fusion technique based on spectral Fourier analysis and Type-I fuzzy classifiers (FFT-Type-I FLC classifier) for the accurate real-time automatic identification and quantification of high-frequency micro-scale HI spike transients (in the gamma frequency band, namely 80–120 Hz, in the latent phase) in high-resolution ECoG recordings, post insult. The article describes the possibility of replacing the CWT block of the previous rbio-WT-Type-1-FLC classifier in [20] with a more straightforward spectral Fourier analysis block that leads to competitive high accuracies compared to the previous wavelet-based technique. The negligible accuracy drop of <1% is within an acceptable marginal error-tolerance range and can be accepted when the algorithm is using a simpler technique. Moreover, the current article provides supplementary spectral analysis materials based on the wavelet power spectral density and pseudo-frequency approximation analysis techniques that mathematically justify the choice of frequency band of 80–120 Hz for HI spike transients and further confirm the correct choice of the mother wavelet and the scale number in our previous work [20]. The complementary spectral material in this article will also further explain the very close performance similarities between the FFT-based approach (in this work) compared to the wavelet-based strategy in the previous work. The very similar performance of the current approach compared to the wavelet-based strategy in our previous work [20] allows the user to choose a strategy based on their computational requirements. Both techniques are expected to perform well if applied to real clinical data, while results demonstrate that the FFT-based approach would be more sensitive to noise. Furthermore, the article addresses beneficial clinical/physiological information around the timing of the injury by reporting where in time the average rate of HI spikes peaks within the latent phase. It will be discussed that a similar trend can be observed for animals with shorter occlusion periods that better mimic clinical situations in the real world. The observation of such a trend in animals with different occlusion periods suggests that high-frequency spike transients can be studied further as another potential biomarker of HIE.

## 2. Related Work

The introduced spectral-based strategy in this work can be compared with a few other experimental and clinical studies focused on the spike, fast ripple, and spike-seizure identification. In the author’s opinion, high-frequency oscillations and fast ripples in the EEG contain the closest spectral features to the high-frequency HI spike transients. In general, the automatic identification and quantification of such fast EEG transients have been discussed to provide beneficial information about the onset of epileptic seizures [23]. Hence, automated schemes have been developed to target the robust identification of these high-frequency transients in the EEG and Magnetoencephalogram (MEG) data. Studies have also demonstrated that the high-frequency scalp oscillations (i.e., interictal fast ripples) in the 250–500 Hz ripple-band can be recorded from the scalp and are detectable using semi-automatic methods based on deep learning strategies with 98% overall sensitivity [27]. Fusion techniques such as the combination of local spectral energy assessments of EEG segments in conjunction with Fourier and wavelet transforms have been shown to be helpful in the automatic classification of fast ripples and interictal epileptic spikes from an EEG background [28]. Research have indicated that high-frequency oscillations (HFO) and epileptic spikes in the ECoG recordings of 3-year-old children can be identified using STFT and time-frequency approaches [29]. Spectral time-frequency analysis have been demonstrated to automatically identify human intracranial HFOs from artefact and other EEG background activity with good specificity of greater than 90% [30].

Researchers have also demonstrated that various techniques based on the combination of spike duration/frequency and autocorrelation along with EEG power can be employed to identify spike seizures in the EEG of asphyxiated adult rats, resulting in acceptable sensitivity and specificity of 100% and 99.98%, respectively [31]. Nonlinear filtering approaches have also been shown to be effective for automatic identification of EEG seizures with different morphologies in immature rat HI models, with 70–80% agreement with manual annotations [32]. A study has demonstrated that the construction of an adapted mother wavelet from an EEG spike template, used in a CWT-based template matching scheme, could improve the identification accuracy of epileptic spike transients to acceptable accuracies of >90% in data from mouse models of epilepsy [33]. Fusion of various techniques such as autocorrelation, wavelet decomposition, and nonlinear energy operator (NLEO) has been shown to also improve the overall sensitivity of spike train seizures detection in more than 217 h of recordings from term neonates [34]. Very recent studies also show that heuristic algorithms can be developed to identify spike trains when the maximums of nonlinear energy components of the signal are compared to the background EEG activity, resulting in an overall good detection rate (GDR) of 95%, tested over 353 h recordings from 81 infants [35]. More recent work has demonstrated that a combinational scheme based on the convolutional neural networks (CNN) and random forest can help to automatically identify neonatal seizures in human babies with 77% overall accuracy [36].

Recent reviews have comprehensively discussed the automated strategies for EEG transient identification in the experimental and clinical data [17,18,37].

## 3. Materials and Methods

### 3.1. Data Acquisition

All the animal data acquisition procedures used in this work were approved by the Animal Ethics Committee of the University of Auckland and in accordance with the Animal Welfare Act (1999) of New Zealand. For consistency with our previous work, and also due to the in utero nature of instrumentation and ethical reasons, the animal sample size of the work was fixed to seven Singleton Romney/Suffolk preterm fetal sheep. A comprehensive description of the data acquisition procedures and surgical protocols have been previously detailed in [20]. HI experiments were conducted on fetuses at around 103–104 days of gestation (0.7 of the full-term gestational period, term = 147 days) to model the preterm human brain at around 27–31 weeks of gestation before the beginning of cortical myelination [11,38]. The fetuses were partially exteriorized for instrumentation. Bipolar recordings from the parietal cortex were obtained through two pairs of EEG electrodes (AS633-5SSF; Cooner Wire, Chatsworth, USA) secured into burr holes created on the dura over the parasagittal parietal cortex (5 and 10 mm anterior to bregma and 5 mm lateral). A reference electrode was also placed over the occiput. Due to the insertion of EEG electrodes on the brain surface, through the skull, this method can be referred to as the ECoG [39,40]. Extradural ECoGs from this space provide superior information and allow capturing higher frequency signal components (i.e., gamma frequency band spectrums) while minimizing noise and artifacts. Fetal heart rate and electrocardiogram (ECG) were measured through additional electrodes secured across the fetus’s chest. Finally, before the fetus was returned to the uterus, an inflatable silicone occluder (In Vivo Metric, Healdsburg, CA, USA) was placed loosely around the umbilical cord. All fetal leads were exteriorized through the maternal flank and a maternal long saphenous vein to provide access for post-operative care and euthanasia.

### 3.2. Post-Surgery Recovery and Data Recording

After the completion of surgery, fetuses were allowed at 4–5 days post-operative recovery before experiments commenced. During this time, welfare monitoring was undertaken several times each day, and ewes received intravenous antibiotics daily for 4 days (benzylpenicillin sodium; 600 mg; Novaris, Auckland, New Zealand and Gentamycin; 80 mg). Fetal asphyxia was induced by complete inflation of the umbilical cord occluder for 25 min (*n* = 4), 19 min (*n* = 1), 15 min (*n* = 2), [7,20,41]. Successful occlusion was confirmed by the rapid onset of bradycardia, a rise in mean arterial blood pressure (MAP), and changes in blood chemistry determined from arterial blood samples [42]. Pre-ductal arterial blood was taken for pH, blood gas (ABL 800, Radiometer, Copenhagen, Denmark), glucose, and lactate measurements (model 2300, YSI, OH, USA) 15 min before occlusion, at 5 and 17 min (where possible) of occlusion, and 2, 4, and 6 h after occlusion. The first 6 h of EEG/ECoG recordings during the recovery period (the latent phase) were examined in this study. The raw ECoG recordings were obtained through electrodes and amplified with a gain of ×10,000. Data was digitized at 1024 Hz for further analysis after being passed through a sixth order low-pass Butterworth anti-aliasing filter and a first-order high-pass filter with cut-off frequencies set at 512 Hz and 1.6 Hz, respectively. Then, the digitized data were extracted and decoded into Matlab for micro-seizure analysis. Initially, the data were zero-meaned and then passed through a 100th-order digital band-pass finite impulse response (FIR) filter with a normalized stop-band frequency (ω) between 0.05 and 0.13 (25.60 Hz < f < 66.56 Hz), if needed. More than 42 h of the raw EEG/ECoG data were analyzed for micro-scale high-frequency HI spike transients.

### 3.3. HI Micro-Scale Transients

The high-frequency micro-scale HI spikes have been fully explained in Section 2.3 of our previous publication [20], and the reader is encouraged to refer to those materials to avoid redundant content. In brief, a conventional EEG spike transient is defined as an event with a duration of less than 70 ms [8,9,10,16,17,20,43]. However, HI spike transients in the ECoG of asphyxiated fetal sheep have been seen to appear in the gamma frequency band (namely 80–120 Hz) with a duration of <20 ms (Figure 1) [8,14,17,18,20,21]. At the micro-scale, the amplitude of the HI transients has been seen to vary from low to moderate magnitudes similar to the subtle waveforms observed in preterm clinical studies [18,44].

For consistency with our previous work [20] and to provide performance comparability, here we considered that high-frequency spike transients with an amplitude of ≥ 14 μV should be annotated. HI spike transients were identified manually by an expert (HA). The database used in this article consists of more than 42 h of 1024 Hz sampled ECoG, collected after a profound occlusion of the umbilical cord, from unanesthetized fetal sheep (*n* = 7). The database includes a total of >3200 spike transients, which is much higher than the total number of reported transients in many other works [45]. A total of 513, 103, 202, 1436, 476, 408, and 153 HI spikes were manually labeled from the first 6 h post HI-insult EEG of 7 fetuses with a range of different occlusion durations of 25, 25, 25, 19, 15, 25, and 15 min, respectively.

### 3.4. Method Description

In this section, we initially detail the application of (1) wavelet power spectral density [46,47] and (2) pseudo-frequency approximation for approximating an optimal spectral frequency range of HI spike transients that best matches a specific mother wavelet. This will later help for the identification of the desired transients (HI spikes) using their spectral characteristics and to design an appropriate spectral filter [48,49,50]. This was followed by introducing fusion techniques using combinations of Fourier analysis and Type-1 FLC classifier [14,20] as well as Fourier with Type-1 FLC classifier. This was done by assessing the results of the application of Fourier transform on the raw EEG and passing the transformed signal into the Type-1 FLC classifier for final reasoning. The article will discuss how the WT section in our previously developed WT-Type-1 FLC classifier, described in [14,20], can be replaced by the Fourier transform section to obtain a combined FFT-Type-1 FLC classifier. To avoid repetitive content, the reader may refer to the citations of the referred methods where needed.

In our previous work [20], we discussed that there is a minimal chance of capturing HI spike transients that appear in the gamma frequency band with the standard clinical sampling rate of 256 Hz. We demonstrated that only the sturdy spikes remain in the signal with lower amplitudes, and there is a small chance to observe many of these transients in the 256 Hz clinical recordings. Therefore, this work will not study or compare the results of the spike transients in down-sampled data (i.e., 256 Hz).

### 3.5. Wavelet Power Spectral Density

**Figure 2:**
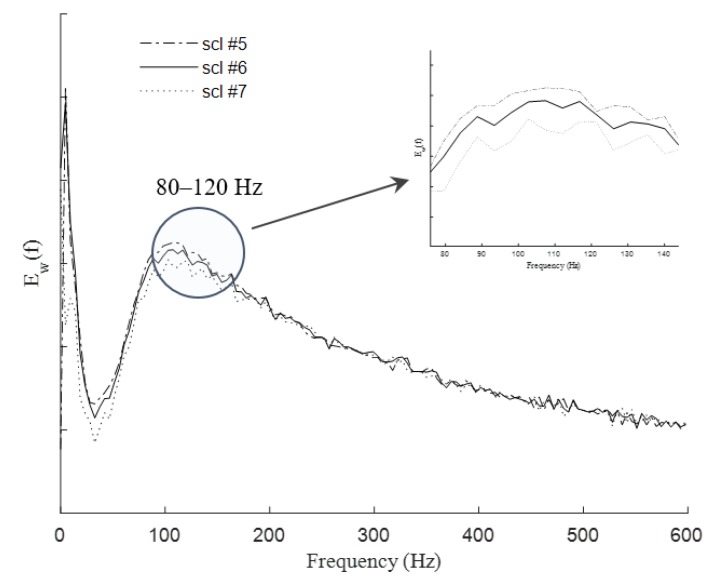
Frequency dependent wavelet energy spectrum using rbio2.8 scales 5–7.

The scale-dependent wavelet energy spectra, *E*(*a*), described in Section 3.1.1 of our previous publication [20], could be converted to a frequency-dependent wavelet energy spectra, *E_w_*(*f*), which is defined as:

(1) $$E_{w}(f)=\frac{1}{f_{c}C_{\varphi }}\int_{0}^{\tau }{\left|T(f,b)\right|}^{2}db$$

where *f_c_* is the scaled band-pass center frequency of the mother wavelet and τ is the length of the sample signal. Peaks in *E_w_*(*f*) demonstrate the dominant energetic frequencies within the signal that are linked to the dominant oscillatory regime of the original transient/signal (spikes in this case) [46]. In other words, such peaks highlight the bandwidths of the dominant spectral components within the desired signal/transient. Therefore, the wavelet power spectral density can represent useful information for identification of a particular event at a specific frequency using a specific scale number. To perform the analysis, the wavelet power spectral density of the HI spike transients was calculated using the suggested optimal mother wavelet (rbio2.8) from our previous work [20] at scales 5, 6, and 7. Figure 2 demonstrates the wavelet power spectral density of the HI spike transient using rbio2.8 at scales 5–7. The peak of the graph suggests that the sample studied HI spike contains an average dominant frequency at 109.4 Hz within the frequency band of 80–120 Hz. In the following section, we will demonstrate how the associated frequency to a particular wavelet scale could be determined using the scaled band-pass center frequency, *f_c_*, of a specific mother wavelet. This will later help to choose a proper band-pass to be used in the Fourier transform.

### 3.6. Pseudo-Frequency Approximation

Pseudo-frequency approximation techniques can be used to specify the spectral information related to a specific pattern (i.e., spike transient here) using a well-matched scale from a particular mother wavelet. In wavelet analysis, it has been shown that the scale value can be related to frequency through determining the central frequency of the basis mother wavelet, *f_c_*. Basically, the spectral components are inversely proportional to the dilation (f∝1/a, *a* = number of scale), and this idea can be used to obtain an approximation for the relationship between the number of the scale and the frequency. The associated frequency with a particular mother wavelet function at a specific scale is called the pseudo-frequency *f_p_* [46,51,52]. Analytically, the band-pass center frequency of a mother wavelet can be used to calculate the pseudo-frequency:

(2) fp=fc(a×Δt),  (Hz)

where *a* represents the scale, ∆*t* is the sampling period (e.g., 1/1024), and *f_c_* is the center frequency associated with the mother wavelet in Hz. Clearly, the center frequency is changed to *f_c_/a* when the wavelet is dilated with parameter *a*. Then, the sampling period, ∆*t*, is used to correspond scale *a* to the pseudo-frequency *f_a_* (Hz). This confirms that significant high-amplitude fluctuations would occur along the cross-correlation coefficients series of an arbitrary signal, which is known as the continuous wavelet transform (CWT), when the pseudo-frequency *f_p_* of the mother wavelet is associated with the local dominant frequencies along the signal. Thus, the information from the formulation above can be used for to determine an appropriate scale for the spectral analysis of a particular waveform at a specific frequency.

Analytical results of the pseudo-frequency calculations, *f_p_*, for the rbio2.8 over a scale range of 1 to 12 using Equation (2) are shown in Table 1. Table 1 highlights that scales 5 to 7 of the rbio2.8 basis wavelet provide a suitable frequency band that best represents the dominant frequency of the spike transients (bolded values in Table 1). In other words, the main frequencies of the oscillations within the scale range of 5 to 7 for rbio2.8 are suggested to be well-matched with the wavelengths corresponding to the length of the HI spikes. From the spectral point of view, Table 1 suggests that rbio2.8 of scales 5 to 7 represent the best spectral correlation to the events with similar associated frequencies in the frequency band of 80–120 Hz (in this case, HI spikes in the gamma frequency band). This confirms that the choice of using a band-pass filter of approximately 80–120 Hz in the Fourier analysis block of the proposed classifier for HI spike identification by capturing the dominant frequency of HI spikes when the Fourier transform is applied to the raw EEG/ECoG signal.

### 3.7. FFT-Type-1-FLC Classifier

The spectral Fourier analysis possesses desirable reconstruction (invertible transformation) capabilities, which allow filtering a specific frequency band from the signal [47]. Building on our recent work in 2019 [20], this work represents a fusion technique based on Fourier analysis and Type-1 FLC classifier for identification of post-HI EEG/ECoG spike transients in the fetal sheep cohort (*n* = 7). In this method, after pre-processing of the original raw signals (zero-meaning and normalization), the high-resolution 1024 Hz EEG/ECoG signals were initially band-pass-filtered through an FFT and then IFFT to time-localize the desired spectral components of the signal that contain a certain frequency range. As described in the Methods section, here we chose the band-pass of 80–120 Hz suggested by the wavelet power spectral density and the pseudo-frequency approximation methods.

**Figure 3:**
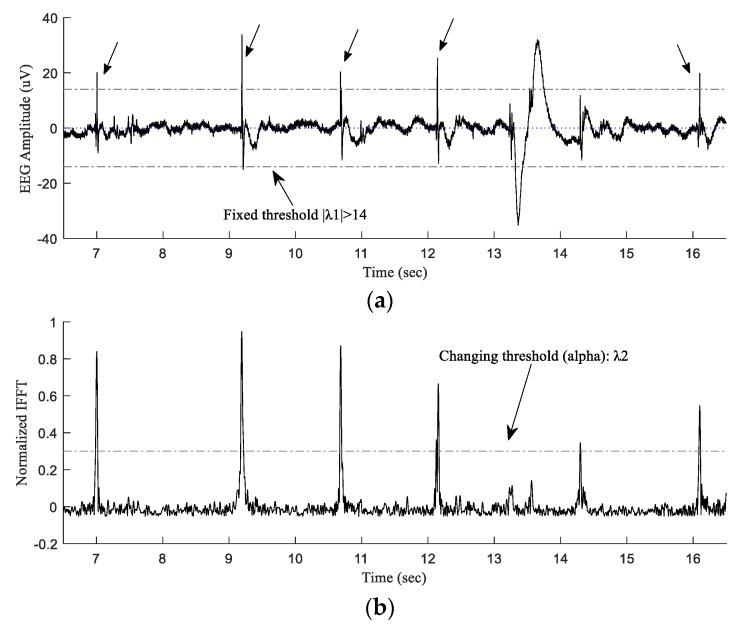
(**a**) A section taken from Figure 1a, spikes are indicated with arrows. (**b**) The inverse FFT of the ECoG signal in (**a**) FFTed over the frequency range of 80–120 Hz. λ_1_ is fixed at 14 *µ*V and λ_2_ (alpha) varies between 0.1 and 0.95.

**Figure 4:**
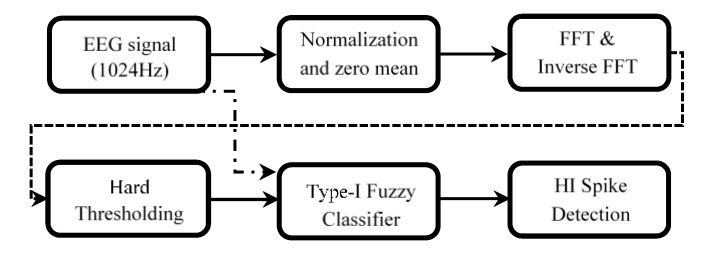
Schematic of micro-scale HI spike transient detector.

Then the output of this stage (inverse FFTed data) along with the raw ECoG signal were passed to the Type-1 FLC classifier block for final decision making. Figure 3 illustrates how the combination of FFT/IFFT and Type-1 fuzzy techniques can be used to develop a spike classifier (FFT-Type-1-FLC classifier). Figure 3a demonstrates a 10-s section of the original 1024 Hz sampled ECoG signal shown in Figure 1, which was obtained from a preterm sheep, post-HI. The post-HI spike transients with different amplitudes are labeled with arrows. The signal was Fourier transformed with a band-pass of 80–120 Hz as suggested by the wavelet power spectral density and the pseudo-frequency approximation analysis before. The inverse FFT signal is shown in Figure 3b. At this stage, both of the signals (the raw ECoG and the inverse FFT transformed signal) are passed to the logical classifier (Type-1 fuzzy) using a fixed and a moving threshold value, λ_1_ and λ_2_, for the raw ECoG and the transformed signal, respectively. This information is used in the decision-making block of the FFT-Type-1-FLC classifier for final reasoning. For instance, for a particular signal point, “if” the original amplitude in the raw signal is either greater or less than 14 *μ*V (|λ_1_| ≥ 14*μ*V) “and” the IFFT signal’s value is either greater or less than the changing threshold value, alpha (|λ_2_| ≥ *alpha*), “then” that particular point in the original ECoG is labeled as a spike transient. The spectral FFT-based analyzing block in the current approach simply allows the desired high-frequency spectrums (within the desired frequency band) to be filtered and passed along to the fuzzy classifier block, whereas this was done by the CWT-based block in our previous wavelet-based approach. Besides that, the results of the wavelet power spectral density and pseudo-frequency approximation analysis in the current article mathematically clarify the correct choice of the reverse biorthogonal mother wavelet and the scale number in our previous work [20]. Such detailed analysis provides robust spectral schemes to expect similar accuracies from the FFT-based approach (in this work) and the wavelet-based strategy in the previous work. The schematic of the proposed HI spike detector is shown in Figure 4. The algorithms were executed in Matlab^®^ on a single workstation computer: Intel^®^ Core™ i7-7700 CPU 3.60 GHz, 4 cores processor with 16 GB RAM memory.

## 4. Performance Measures

For performance assessments of the algorithm, sensitivity and selectivity measures of (*TP* × *100*)/(*TP* + *FN*)% and (*TP* × *100*)/(*TP* + *FP*)% were evaluated, respectively, and the overall performance was obtained by the evaluation of the average value of sensitivity and selectivity for different values of λ_2_ [14,20]. The precision–recall curves (PRC) [53] results of the proposed algorithm are plotted using the sensitivity (recall) and selectivity (precision) measures obtained at different λ_2_s. The PRC provides a graphical representation of how the overall performance of the algorithm progresses for a changing λ_2_. A PRC trend moving toward the upper right-hand corner is highly aimed and represents a much better algorithm performance with a lower number of false detections.

## 5. Results

**Figure 5:**
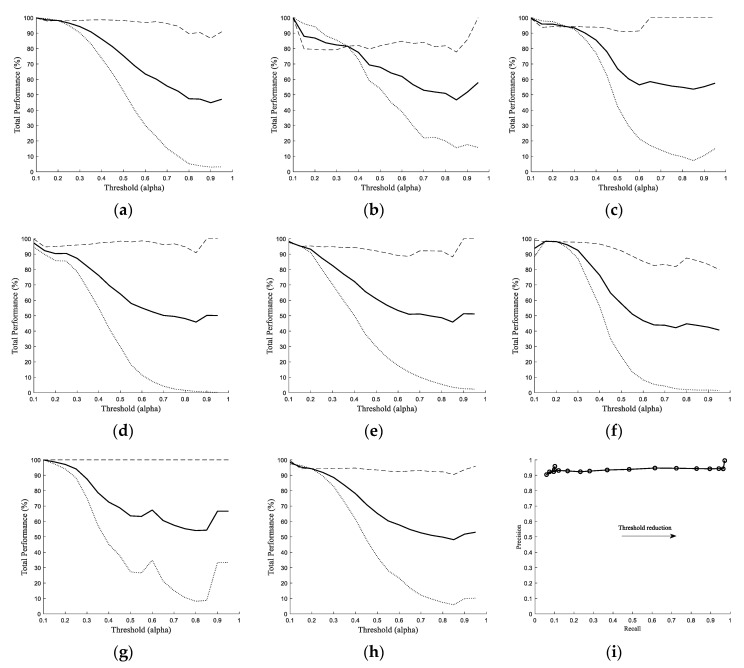
Fourier analysis and Type-I fuzzy classifiers (FFT-Type-1 FLC) classifier performance over different λ_2_ thresholds for seven fetal sheep (sheep **a**–**g**). Band-pass: 80–120 Hz. Dotted lines: sensitivity, dashed lines: selectivity, bold lines: overall performance. (**h**) is the overall performance of the classifier for 7 sheep. (**i**) Precision–recall curve showing the overall performance.

**Figure 6:**
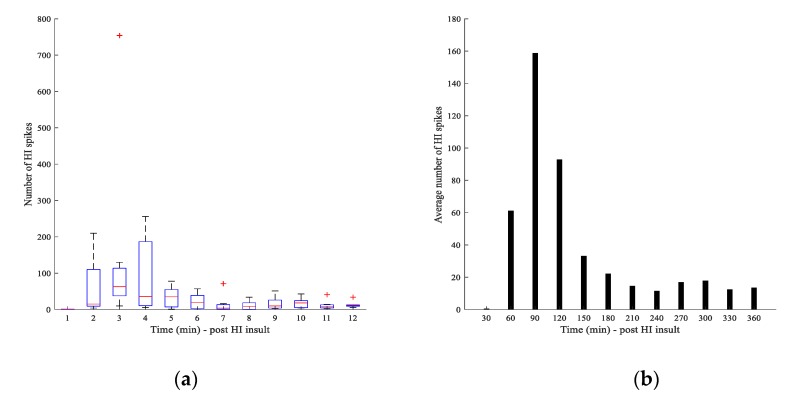
(**a**) Boxplot of the TP hits by the FFT-Type-1-FLC classifier in 30-min epochs within the latent phase, post-HI insult. (**b**) Average number of TP hits by the proposed classifier in 30-min epochs within the latent phase, post-HI insult.

The performance of the FFT-Type-1-FLC classifier for identification of post-HI spike transients (in the gamma frequency band) was evaluated for different λ_2_s using the latent phase ECoG from seven preterm fetal sheep (total of 42 h data). Figure 5a–g demonstrate the results of the proposed classifier for each individual animal. Figure 5h represents the overall PRC results of the FFT-Type-1 FLC classifier approach versus different λ_2_ threshold values for seven different fetal sheep. In Figure 5a–g, dotted and dashed lines represent the sensitivity and selectivity, respectively, and the overall performances are shown with continues lines. The maximum overall performances from each sheep were calculated as 99.90%, 99.03%, 99.75%, 97.24%, 97.96%, 98.52%, and 100.00% for sheep A to sheep G, respectively (Table 2). A mean overall performance of 98.87 ± 1.00% was obtained from the FFT-Type-1-FLC classifier confirming the reliability of this spectral approach for identification of post-HI spike transients in 1024 Hz ECoG data. Table 3 represents the total number of true-positive (TP) and false-positive (FP) hits by the automatic FFT-Type-1-FLC classifier over 6 h of latent phase data from each sheep (at the best-evaluated threshold value), post HI. The results of Table 3 demonstrate an extremely good correlation with the results of our previous wavelet-based approach with confidence values of *p* < 0.0001 and *r* > 0.99. Table 3 shows that the highest overall number of quantified post-HI spike transients occur between 1 and 2.5 h (60–150 min) after release of umbilical cord occlusion in animals with different occlusion periods. The average number of correct detections (TPs) by the FFT-type-1 FLC classifier over the best threshold value as well as the corresponding box-plot, using 30-min bins in the latent phase of all animals, are demonstrated in Figure 6. This is promising as approximately 90 min post-reperfusion (or 2 h from the beginning of the HI insult) is when the administration of cerebral hypothermia has been shown to be optimally effective against the spread of injury in near-term fetal sheep [12].

Compared to the “no false-negative detection” results of the WT-Type1-FLC classifier reported in the previous work [20], the FFT-Type-1-FLC classifier resulted in 99 false-negative detections (missed hits) among more than 3200 spike transients from seven animals (see Table 3). There was no big change observed in the total number of false-positive hits (wrong detections) by the FFT-Type-1-FLC classifier compared to the WT-Type1-FLC method. In our previous work [20], we demonstrated that the wavelet-only method did not result in acceptable performances. Therefore, due to the similarity between the wavelet-only and Fourier-only methods, this work did not further investigate the performance of the spectral-only method. Compared to the previously reported overall performance of 99.78 ± 0.10% for the wavelet-based strategy (WT-Type1-FLC) [20], the application of the spectral-based analysis method (FFT-Type-1-FLC) resulted in an almost 1% reduction in the overall performance over the entire preterm fetal sheep cohort (*n* = 7).

## 6. Discussion

The latent phase after HI injury is considered as the most critical period for the detection of EEG transient biomarkers for HIE. Early identification of these subtle EEG markers could provide the earliest opportunity to diagnose the injury and initiate possible neuroprotective treatments before the window of opportunity is closed. This work has introduced a successful alternative spectral-based approach with competitive high accuracy compared to our previous wavelet-based technique. The method offers real-time automatic identification of post-HI EEG spike transients that appear in the gamma frequency band (80–120 Hz) during the latent phase. The work is a complementary extension to our previously developed wavelet-based HI spike detector, the WT-Type-1-FLC classifier [20], demonstrating that the simpler computational characteristics of the spectral Fourier analysis block could desirably make it an alternative choice to the wavelet-based analysis block in the previous work. The spectral Fourier analyzing block in the current approach simply allows the desired high-frequency spectrums within the defined frequency band to be chosen and then passed along to the fuzzy classifier, while the CWT-based block in the previous WT-Type-1-FLC approach was responsible for initially filtering the high-frequency EEG components. The proposed FFT-Type-1-FLC classifier was able to accurately identify and quantify high-frequency micro-scale spike transients with competitive high accuracies compared to the WT-Type-1-FLC technique. An overall performance of 98.87 ± 1.00% was obtained for the FFT-Type-1-FLC classifier over 2520 min of latent phase recordings including ≥3200 spike transients from seven fetal sheep, with different occlusion lengths including 25 min (*n* = 4), 19 min (*n* = 1), and 15 min (*n* = 2). The performance of the FFT-Type-1-FLC classifier was consistent between individual animals as well as for the entire cohort. The negligible accuracy drop of <1% is within an acceptable marginal error-tolerance range and can be accepted given that the algorithm is computationally simpler.

The spectral band-pass frequency for FFT analysis was initially investigated through (1) wavelet power spectral density and (2) pseudo-frequency approximation analysis techniques, confirming that the frequency band of 80–120 Hz was suitable for further analysis of HI spike transients. In addition, the findings from spectral analysis in the current paper confirm the correct choice of mother wavelet and the scale number in our previous work and justify the very close performance similarities between the FFT-based and the wavelet-based strategies. Compared to the WT-Type-1-FLC classifier in [20], the FFT-Type-1-FLC classifier was observed to be more sensitive to the variations in the alpha threshold parameter, resulting in much lower performances for an increasing alpha (the reader is encouraged to compare the represented results in Figure 8a of reference [20] with Figure 5h of the current article). The very close high-performance results obtained from the introduced spectral-based approach in this article compared to the previous wavelet-based strategy allow the user to choose a technique based on their computational requirements and needs. We expect both strategies to perform well for transient identification in real (and challenging) clinical data, while the results demonstrate that the spectral-based approach would be more sensitive to noise.

Since the technique introduced in the current article is generic, it would be a reliable choice for any similar pattern recognition application, including the identification and quantification of other types of HI transients in clinical recordings. Furthermore, the automatic quantification results of the time-localized high-frequency micro-scale HI spike transients demonstrated a promising trend about where exactly in time the average number of these transients peak, post insult. Considerably higher numbers of HI spike transients were automatically quantified within the first 2 h post-HI. Trends with similar behavior were observed in animals with shorter occlusion periods, where the length of occlusion is milder and likely to be closer to real clinical situations. This emphasizes that the high-frequency spike transients need to be studied further as potential robust biomarkers of HIE. It is believed that such patterns reveal clinically relevant information about the physiological aspects of the HIE and therefore are of high interest for studies of HI. For instance, preterm animal models of HI have proven that cerebral hypothermia can lead to optimal outcomes if the treatment is initiated at 90 min post-reperfusion [11,12]. This time window is consistent with the finding of the current paper, as the number of automatically quantified spike transients peaked at 60–120 min post-HI. These observations emphasize the necessity of research to further investigate any potential correlations between the time-localized micro-scale HI gamma spikes and histological outcomes. The findings also support the necessity of upgrading the current low 256 Hz clinical sampling frequency to higher rates that will allow clinicians to acquire detailed embedded information during recordings at different stages of the evolution of HIE.

## Figures and Tables

**Table 1:** Scale number, pseudo-frequency approximation, and wavelength of the rbio2.8 mother wavelet over the scale range of 1–20.

Scale Number	Pseudo Freq. (Hz)	Wavelet Length (ms)
1	602.491	1.700
2	301.246	3.399
3	200.830	5.099
4	150.623	6.798
**5**	**120.498**	**8.498**
**6**	**100.415**	**10.198**
**7**	**86.070**	**11.897**
8	75.311	13.597
9	66.943	15.296
10	60.249	16.996
11	54.772	18.696
12	50.208	20.395

**Table 2:** Performance of the FFT-type-1-FLC classifier using the band-pass frequency of 80–120 Hz indicated for the best threshold value.

Sheep No.	a	b	c	d	e	f	g
Maximum Tot. Performance (%)	99.90	99.03	99.75	97.24	97.96	98.52	100.00
At Threshold	0.10	0.10	0.10	0.10	0.10	0.15	0.10

**Table 3:** Performance records (TP, FP, and FN hits) of the FFT-Type-1 Fuzzy classifier over the best threshold value in seven preterm fetal sheep. TP: true positive, FP: false positive, FN: false negative.

Sheep No.		Sheep a			Sheep b			Sheep c			Sheep d			Sheep e			Sheep f			Sheep g		
Occlusion Length (min)		25			25			25			19			15			25			15		
	Detection Type:	TP	FP	FN	TP	FP	FN	TP	FP	FN	TP	FP	FN	TP	FP	FN	TP	FP	FN	TP	FP	FN
Time—Post HI (min):																						
30 (reperfusion phase)		0	1	0	1	0	0	1	0	0	1	0	0	0	0	0	0	0	1	0	0	0
60		15	0	0	4	0	0	10	0	0	131	0	1	210	3	8	9	1	0	48	0	0
90		58	0	0	31	1	0	130	0	0	754	0	2	63	0	0	65	0	0	10	0	0
120		118	0	0	13	0	0	10	1	0	210	0	0	36	1	2	256	0	4	6	0	0
150		59	0	0	7	0	0	1	0	0	78	0	0	35	2	0	43	0	3	8	0	0
180		57	0	0	8	0	0	1	0	0	44	0	0	24	0	1	0	0	0	20	0	0
210		71	0	0	4	0	0	0	0	0	16	0	0	4	0	0	0	0	0	6	0	0
240		34	0	0	3	1	0	0	0	0	14	0	0	20	1	0	0	0	0	9	0	0
270		51	0	0	3	0	0	7	0	0	14	0	11	30	0	1	10	0	1	3	0	0
300		25	0	0	6	0	0	18	0	0	43	1	10	23	0	0	4	0	0	5	0	0
330		14	0	0	10	0	0	9	0	0	41	1	5	6	0	0	2	1	1	4	0	0
360		10	0	0	11	0	0	13	0	0	11	0	48	6	0	0	9	0	0	34	0	0
